# Nuclear Alpha-Synuclein in Parkinson's Disease and the Malignant Transformation in Melanoma

**DOI:** 10.1155/nri/1119424

**Published:** 2025-01-06

**Authors:** María E. Jimenez-Capdeville, Erika Chi-Ahumada, Francisco García-Ortega, Juan Pablo Castanedo-Cazares, Robert Norman, Ildefonso Rodríguez-Leyva

**Affiliations:** ^1^Departamento de Bioquímica, Facultad de Medicina, Universidad Autónoma de San Luis Potosí, San Luis Potosi, Mexico; ^2^Coordinación Académica Región Altiplano, Universidad Autonóma de San Luis Potosi, Matehuala, San Luis Potosí, Mexico; ^3^Departamento de Dermatología, Hospital “Ignacio Morones Prieto”, San Luis Potosí, Mexico; ^4^Center for Geriatric Dermatology, Integrative Dermatology and Euro-Dermatology, Tampa, Florida, USA; ^5^Departamento de Neurología, Hospital “Ignacio Morones Prieto”, San Luis Potosí, Mexico

## Abstract

**Background:** Alpha-synuclein (ASyn), a marker of Parkinson's disease (PD) and other neurodegenerative processes, plays pivotal roles in neuronal nuclei and synapses. ASyn and its phosphorylated form at Serine 129 (p-ASyn) are involved in DNA protection and repair, processes altered in aging, neurodegeneration, and cancer.

**Objective:** To analyze the localization of p-ASyn in skin biopsies of PD patients and melanoma.

**Methods:** Biopsies from 26 PD patients, 20 melanoma patients, and 31 control subjects were probed and analyzed with a p-ASyn antibody by immunohistochemistry and immunofluorescence. Nuclear positivity was quantified by image analysis.

**Results:** Peripheral nerve endings from healthy subjects show little p-ASyn immunopositivity but notable axonal presence in PD. Control subjects show immunopositivity to p-ASyn along all epidermic strata and scarce presence in their cytoplasm. In contrast, its nuclear presence in PD is weaker, with a higher cytoplasmic and intercellular presence. Nuclear p-ASyn in melanoma varied from similar to control skin in early stage melanoma to a higher rate of empty nuclei in the intermediate stage and total absence of nuclear p-ASyn in severe cases.

**Interpretation:** These findings support the nuclear localization of p-ASyn in skin cells and show that its presence decreases PD and almost disappears in the malignant transformation of melanocytes, redistributing to the cytoplasm and intercellular spaces. This confirms the association between PD and melanoma, providing crucial insights into the role of p-ASyn in both diseases.

**Trial Registration:** ClinicalTrials.gov identifier: NCT01380899.

## 1. Introduction

The importance of alpha-synuclein (ASyn) as a neuronal protein arises from its role in Parkinson's disease (PD) and other fatal neurodegenerative disorders. ASyn is the main component of insoluble aggregates that spread in a prion-like manner within brain cells as the disease progresses. Misfolded ASyn presence in cell inclusions termed Lewy bodies or Lewy neurites correlates with the gradual death of affected neurons [1, 2]. Since the predominant ASyn species in Lewy bodies is its phosphorylated form at Serine 129 (p-ASyn) [3], an essential role of p-ASyn has been suspected in PD pathophysiology, although the mechanism is still unclear.

For more than a decade, evidence of ASyn presence in peripheral tissues showing a significant association with PD has been reported. ASyn has been revealed in peripheral nerve terminals [4], skin biopsies [5], the enteric nervous system [6], olfactory bulb cells [7], and saliva [8]. The skin has become an essential target of PD research since ASyn presence could also be related to the nonmotor PD manifestations and associated diseases such as dysautonomia, seborrheic dermatitis, sweating disorders, bullous pemphigoid, and rosacea [9]. Moreover, an assay developed to measure the aggregation capacity of ASyn isolated from skin biopsies allows a clear distinction between PD patients and healthy subjects, leading to a reliable biomarker for PD [10].

Recent discoveries about neurodegeneration show that the complete pathological PD phenotype cannot be explained only by ASyn aggregation and spreading [11, 12]. Though misfolded ASyn is neurotoxic, the loss of normal ASyn protein functions as it aggregates disturbs synaptic communication, energy generation, response to oxidative stress, and DNA protection, which are crucial components of the disease [13, 14]. Protecting functions have been attributed to ASyn expression in other cell types, such as melanocytes [15] and fibroblasts [16]; notably, ASyn is highly expressed in melanoma [17, 18], a cancer type with epidemiological, genetic, and risk factor coincidence points with PD [19]. Protective ASyn functions may allow melanoma cells' fast growth and survival upon a high ASyn expression [20]. In this respect, silencing ASyn expression significantly affects melanoma growth [21, 22].

The long-standing view of ASyn as a synaptic protein disregarded the importance of its nuclear localization [23, 24]. ASyn and p-ASyn are also located in cell nuclei and involved in DNA binding and repair [14, 25]. These functions suggest a role in altered gene expression that could contribute to neurodegenerative processes and malignant transformation in melanoma [11, 26]. The recent research indicates that there are shared characteristics between neurodegeneration and cancer, such as their essential association with aging and DNA damage. The overlap between seemingly opposite processes, one leading to cell proliferation and the other to cell death, may involve ASyn in the case of PD and melanoma.

In light of this, we hypothesized that a decreased function of nuclear p-ASyn could be at the core of altered gene expression in skin cells undergoing PD or melanoma. Our objective was to analyze the distribution of p-ASyn in PD skin and melanoma because p-ASyn is the main constituent of Lewy bodies [3, 27], and its presence in the peripheral nerve terminals of PD patients has been proposed as a biomarker of the disease [28].

## 2. Materials and Methods

### 2.1. Participants

Four mm punch biopsies from retro auricular skin were obtained from 26 PD patients and 31 age- and sex-matched neurologically healthy individuals who agreed to participate in the study (Table 1). The Queen's Square Brain Bank criteria were employed for PD diagnosis, and all participants signed informed consent. The local Ethics and Research Committee evaluated and approved the investigation protocol and the consent with the UASLP-PD001 registration number. Twenty melanoma biopsies were obtained from the Center for Geriatric Dermatology, Integrative Dermatology, and Neuro-Dermatology in Tampa, Florida. The depth and dissemination of malignant cells were expressed according to Clark's level, which ranged in these biopsies from 1 to 4, from the presence of transformed cells only in the dermis in Level 1 biopsies to the complete spreading of other tissues in Level 4.

### 2.2. Immunohistochemistry and Confocal Microscopy

Skin biopsies were fixed in 4% buffered paraformaldehyde, dehydrated and embedded in paraffin blocks. For immunohistochemistry, 5 μm tissue slices were collected on electrocharged slides (Biocare Medical LLC, Concord, CA) for dewaxing and rehydration by xylene and ethanol rinses. Epitope recovery was performed in Diva Decloacker solution (Biocare Medical LLC, Concord, CA) in a pressure cooker, followed by endogenous peroxidase depletion by incubating them in 3% H_2_O_2_. Then, tissue samples were incubated for 15 min with a nonspecific background staining blocker (Background sniper, Biocare Medical LLC, Concord, CA) and endogenous biotin and biotin-binding protein blocker (avidin/biotin blocking kit; Vector Laboratories, Inc., Burlingame, CA) alternating with Tris-buffered saline plus Tween-20 (TBST) rinses. The rabbit anti-29 phosphorylated ASyn antibody (ABCAM, ab59264 dilution 1:100) was incubated overnight, followed by 30 min with a streptavidin–biotin detection system (Dako Solutions). Peroxidase activity was visualized by incubating the sections with amino ethyl carbazole for 9 min to obtain a positive red color. Samples were counterstained with Harris hematoxylin. Photomicrographs were obtained in a light microscope with a digital camera (Olympus CX31). Five fields per sample were captured at 40x magnification. They were digitally analyzed using an ad hoc automatized image segmentation algorithm that counts the number of positive (red nuclei) and blue nuclei, indicating the absence of p-ASyn [29]. Although a complete color range from blue to red was observed in the skin sections, according to the amount of nuclear p-ASyn, with the purpose of quantitative comparison, a threshold of red and blue colors based on RGB analysis was established as a marker of presence and absence of p-ASyn, respectively.

For confocal microscopy, samples were processed as mentioned for immunohistochemistry, except for using peroxidase and biotin blockers. Following antigen recovery with Diva Decloacker solution and background sniper (see above), 5 mm skin slices were incubated with the primary antibodies: mouse anti-ASyn (BD Biosciences) dilution 1:100 and rabbit antidilution 1:100 overnight at 4°C; the secondary antibodies were goat anti-mouse IgG marked with Alexa Fluor 633 (dilution 1:100) and goat anti-rabbit IgG marked with Alexa 488 (dilution 1:200) incubated for 2 h at room temperature and light-protected. Nuclei were visualized with nucleic acid stain Sytox (Molecular Probes). The slides were covered using the mounting solution VectaShield. Samples were analyzed for fluorescence in a confocal microscope (LEICA TCS SP2, Leica Microsystems GmbH) at 40x magnification.

### 2.3. Statistical Analysis

The number of p-ASyn positive nuclei for each biopsy was obtained using an automatized image segmentation algorithm [29]. Data were collected using the nonparametric multicomparison test, Kruskal–Wallis, followed by Dunn's test. The statistical significance was set at a *p* value of 0.05 or higher. The analysis and graphics were performed in GraphPad Prism 5 software.

## 3. Results

### 3.1. p-ASyn has a Predominant Nuclear Expression in the Skin

**Figure 1:**
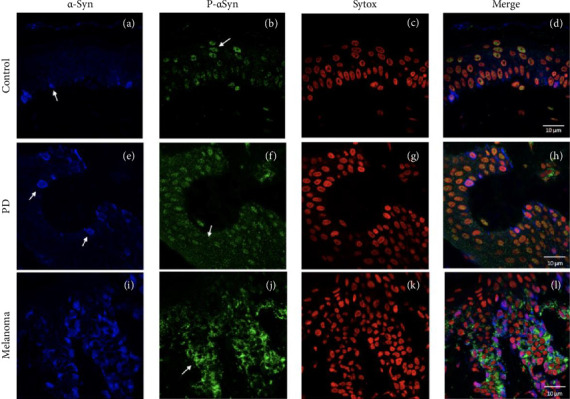
Comparison between ASyn and p-ASyn immunopositivity in skin biopsies. (a) Cyanine 5 (blue) shows the predominant cytoplasmatic localization of ASyn in basal cells and some keratinocytes. (b). Nuclear and perinuclear p-ASyn (Alexa fluor 488, green) across all epidermis strata and scarce ASyn p-ASyn immunopositivity overlapping in healthy skin (d). PD skin reveals higher ASyn presence (e) and the intra- and intercellular spreading of p-ASyn immunopositivity (f, h, merged image). Melanoma cells display high ASyn presence (i), but p-ASyn is scarcely present in the cell nuclei (j). The merged image shows the intercellular spreading of ASyn and p-ASyn (l)—scale bar 10 μm, (c, g, and k), nuclei staining with Sytox.

Both ASyn and p-ASyn are expressed in different cell types in human skin. While ASyn immunopositivity is mainly located in the cytoplasm of melanocytes in the stratum basale, as previously reported (Figure 1(a), arrow), the nuclei of skin cells of control subjects show immunopositivity to p-ASyn along all epidermic strata, and scarce scattered presence in their cytoplasm (Figure 1(b)). The merged image (F1D) presents minor colocalization (yellow marks) of both antibodies in some basal nuclei. In contrast, more intense and extended immunopositivity to ASyn (Figure 1(e)) was observed in the nuclei and cytoplasm of the PD epidermis. Concerning p-ASyn, although nuclear presence seemed lighter than in control subjects, abundant cytoplasmic presence was observed in all skin strata (Figure 1(f)). The merged image shows the nuclear presence of ASyn (Figure 1(h), pink nuclei), and since the nuclear presence of p-ASyn is weaker than in control skin, no colocalization with ASyn was observed in PD basal cells. The melanoma cases showed a higher expression of ASyn (Figure 1(i)). The most striking difference was the total absence of p-ASyn in the nuclei of all kinds of epidermis cells, simultaneously with a higher cytoplasmic and intercellular presence (Figures 1(j) and 1(l)).

### 3.2. p-ASyn Nuclear Presence in Peripheral Nerve Terminals

**Figure 2:**
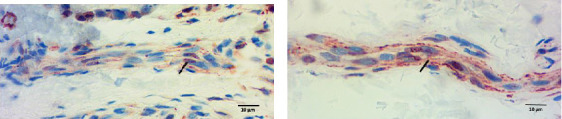
p-ASyn presence in peripheral nerves evidenced by immunohistochemistry. (a) In a control subject, p-ASyn is primarily present in the nuclei of Schwann cells (arrow). (b) In a PD patient, the axons composing the nerve tract have a rich p-ASyn immunopositivity (arrow), while nuclei are scarcely stained. Aminoethyl carbazole staining; scale bar 10 μm.

**Figure 3:**
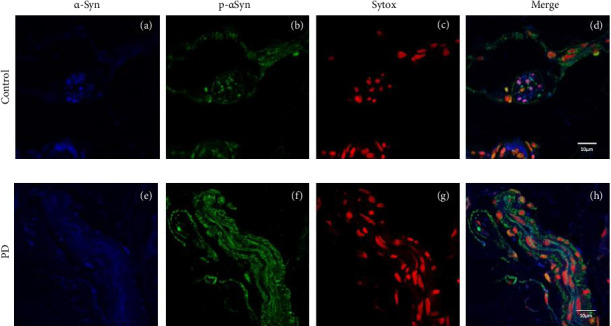
Confirmation of abundant p-ASyn presence in peripheral nerve terminals of PD skin compared to healthy skin. Asyn (blue) and p-ASyn (green) are primarily present in the cell nuclei of Schwann cells and scarcely in the axon path (a, b). The merged image shows pink nuclei with green dots due to the presence of both ASyn forms (d). PD nerve terminal has higher axonal expression of ASyn (e) and p-ASyn (f). Cell nuclear expression in PD (c, g). In the merged image (h), the higher presence of both ASyn forms is extranuclear along the axonal. Scale bar 10 µm.

Peripheral nerves from healthy subjects show scarce p-ASyn immunopositivity (Figure 2(a)), as compared with the noticeable axonal p-ASyn immunopositivity that has been widely reported. Nevertheless, it is worth mentioning the nuclear presence of p-ASyn in Schwann cells of peripheral nerve terminals of control skin (Figure 2(a)). At the same time, in PD biopsies, p-ASyn spreading through the axoplasm is accompanied by p-ASyn empty nuclei (Figure 2(b)). This phenomenon was further explored using immunofluorescence, where ASyn (blue) and p-ASyn (green) nuclear presence is evident in a transversal section of a control skin (Figures 3(a) and 3(b)) and the merged image (Figure 3(d)). By contrast, the longitudinal section from a nerve of a PD patient shows a lower presence of nuclear ASyn (Figure 3(e)) and p-ASyn (Figure 3(f)). Again, the axonal immunopositivity of p-ASyn is very noticeable in the PD nerve, as compared with ASyn, as was demonstrated with immunohistochemistry.

### 3.3. p-ASyn Leaves the Cell Nucleus in PD and Melanoma

**Figure 4:**
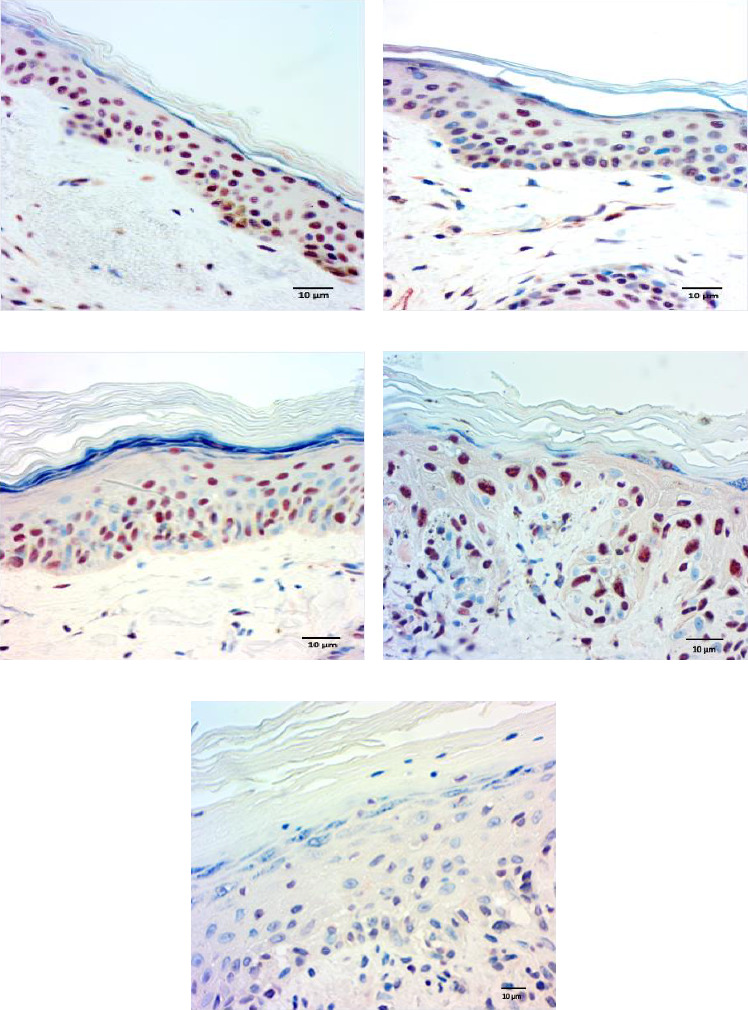
p-ASyn expression in the epidermis evidenced by immunohistochemistry (aminoethyl carbazole staining). (a) Exclusive nuclear presence along all strata of the healthy epidermis (red nuclei). (b) Heterogeneous nuclei in PD epidermis, empty (blue), semioccupied (purple), and full (red) p-ASyn immunopositivity nuclei. Positivity progressively disappears from cell nuclei as malignant melanoma severity increases. (c) Stage 1 melanoma, (d) Stage 3 melanoma, and (e) Stage 4 melanoma. Scale bar 10 μm.

The quantification of nuclear p-ASyn in dermis cells revealed positive nuclei in control biopsies between 20% and 60% (IQ range: 21.3–63) (Table 1). A high dispersion of values in PD patients was observed, where some biopsies showed very scarce positive nuclei (Figures 4(a) and 4(b), Table 1). Melanoma values for nuclear p-ASyn ranged from similar values to control skin in early stages melanoma, a higher rate of empty nuclei in the intermediate stage, and total absence of nuclear p-ASyn in severe cases (Figures 4(c), 4(d), and 4(e)). Therefore, quantifying nuclear p-ASyn in dermis cells revealed positive nuclei in control biopsies, which is lower for PD and even more for melanoma (Table 1).

These results support the nuclear localization of p-ASyn in skin cells and show that its presence decreases PD and malignant transformation of melanocytes, redistributing to the cytoplasm and intercellular spaces.

## 4. Discussion

The well-established direct proportional relationship between melanoma and PD involves both genetic and environmental factors, in addition to occurring more frequently with aging, which they share with other neurodegenerative diseases and other cancers. Oxidative damage to DNA is associated with diverse factors such as cellular metabolism, exposure to toxic agents, diet, or UV radiation accumulation as age increases [30]. Mesencephalic dopaminergic cells and skin melanocytes express either neuromelanin or melanin, respectively, to neutralize the effects of free radicals [31, 32]. Notably, ASyn is highly expressed in both cell types to activate oxidative defenses [15, 33] and to participate in DNA repair [14]. It has been demonstrated that ASyn is recruited to sites of double-strand breaks (DBS) to mediate DNA repair in human cell lines, mouse models, and primary cell cultures. Neurons from ASyn-KO mice and human neurons containing Lewy bodies show increased DNA damage, which confirms direct ASyn participation in DNA repair [14, 34].

Human skin has emerged as a PD model that allows the study of pathogenic mechanisms [35] and therapeutic approaches, providing a reliable disease biomarker [9, 10]. p-ASyn images of spreading in the dermis and peripheral nerve terminals are reported here, as well as the previously demonstrated ASyn spreading in the skin [18], reflecting brain pathology. A defining characteristic of PD and other neurodegenerative diseases is the presence of cytosolic deposits and the spreading of misfolded proteins, which have been suspected to be the cause of progressive neuronal dysfunction and death. Nevertheless, the extensive unsuccessful results of targeting misfolded proteins to cure these diseases or to modify their temporal course have relieved the culprit of proteinopathy as the leading cause of neurodegeneration. The recent research, in contrast, reveals that ASyn, tau, and TDP43 [36, 37], the proteins whose cytosolic accumulations are present in most neurodegenerative diseases, play an essential role as nuclear proteins in healthy cells participating in the modulation of gene expression.

ASyn is present both in the cytoplasm and nucleus of neurons and melanocytes. It is involved in dopaminergic vesicle traffic and the melanosome transfer from melanocytes to keratinocytes. Although neuronal ASyn aggregation in Lewy bodies and Lewy neurites represents a loss of function related to neurodegeneration, the decrease of ASyn protection from oxidative damage and DNA repair is more closely associated with neuronal death. Aging is a leading risk factor for PD since, after prolonged cellular damage, neurons attempt to reenter mitosis to activate their DNA repair mechanisms [38]. Since neurons are terminally differentiated cells unable to divide, this aberrant cell-cycle reactivation leads to neuronal dysfunction and death [39]. In this respect, the progressive loss of mesencephalic dopaminergic neurons is accompanied by markers of DNA oxidative damage, changes in gene expression, and the loss of genome protection by ASyn and p-ASyn when they aggregate in the cytoplasm [13, 26, 40].

Melanocytes require ASyn for survival and pigment transfer [20]. Recent experiments suggest that ASyn helps evade the inflammatory response mounted to limit its proliferation in melanoma malignant transformation, achieved through direct transcriptomic modulation by ASyn [41]. A decrease of ASyn presence in the skin is reported in skin biopsies of relapsing patients with multiple sclerosis [42] and the knockdown of ASyn melanoma proliferation [21]. These phenomena underlie the participation of ASyn in neurodegeneration and melanoma, which can be regarded as divergent results of the same underlying process, namely, aberrant cell-cycle activation trying to restore homeostasis.

The present findings further support an association between PD and melanoma through ASyn by demonstrating changes in its phosphorylated form in skin cells in both diseases. In this respect, most p-ASyn studies focus on its expression along peripheral nerve tracts and central neurons [25, 26, 43], but its nuclear presence in keratinocytes has yet to be reported. Our results agree with previous studies showing p-ASyn immunopositivity expanding along the axoplasm of peripheral nerve terminals; this implies that positivity within keratinocytes and Schwann cells in skin samples is a true positive mark. Since phosphorylation increases ASyn aggregation, these images of extranuclear p-ASyn support the higher seeding activity of ASyn in PD skin, which has been proposed as a disease biomarker. Furthermore, the spreading of p-ASyn revealed in melanoma specimens strongly agrees with the intercellular release of p-ASyn observed in cell cultures of melanoma cells [17]. We propose that the loss of nuclear p-ASyn observed in PD skin biopsies and melanoma cells contributes to inefficient DNA repair, accumulating DNA damage, gene transcription alterations [12], and abnormal cell-cycle reactivation [39].

### 4.1. Summary and Perspectives

Genetic traits and environmental factors that damage DNA, such as exposure to toxic agents, diet, and UV radiation, accumulate throughout aging. These factors overwhelm repair mechanisms and finally modify the gene expression pattern and functionality of mesencephalic dopaminergic neurons and skin melanocytes. These alterations propitiate either a degenerative (PD) or neoplastic (melanoma) pathology. Applying protective, preventive, and curative measures could modify the disease, achieve nuclear DNA repair, and allow cell-cycle normalization in the central nervous system and the skin.

## Figures and Tables

**Table 1:** The quantification of nuclear p-ASyn in dermis cells revealed positive nuclei in between 20% and 60% of control subjects' biopsies (IQ range: 21.3–63), lower for PD and even more for melanoma.

	Control	PD	Melanoma
*N*	32	26	20
% p-ASyn positive nuclei (median)	35	30.5	13.5⁣^∗^
IQ range	21.3–63	4.8–42	3–45.3

⁣^∗^*p* < 0.05 Kruskal–Wallis followed by Dunn's multiple comparison test. See text for algorithm description for the calculation of % positive nuclei.

## Data Availability

The data that support this study's findings are available from the corresponding author upon reasonable request.
